# Insufficient β-lactam concentrations in the early phase of severe sepsis and septic shock

**DOI:** 10.1186/cc9091

**Published:** 2010-07-01

**Authors:** Fabio Silvio Taccone, Pierre-François Laterre, Thierry Dugernier, Herbert Spapen, Isabelle Delattre, Xavier Wittebole, Daniel De Backer, Brice Layeux, Pierre Wallemacq, Jean-Louis Vincent, Frédérique Jacobs

**Affiliations:** 1 Department of Intensive Care, Erasme Hospital, Université Libre de Bruxelles, route de Lennik 808, 1070 Bruxelles, Belgium; 2 Department of Intensive Care, Cliniques Universitaires St-Luc, Ave Hippocrate 10, 1200 Brussels, Belgium; 3 Department of Intensive Care, St-Pierre Hospital, Avenue Reine Fabiola 9, 1340 Ottignies, Belgium; 4 Department of Intensive Care, Universitair Ziekenhuis Brusse, Laarbeeklaan 101, 1090 Brussel, Belgium; 5 Department of Clinical Biochemistry and Pharmacokinetics, Cliniques Universitaires St-Luc, Ave Hippocrate 10, 1200 Brussels, Belgium; 6 Department of Infectious Diseases, Erasme Hospital, Université Libre de Bruxelles, route de Lennik 808, 1070 Bruxelles, Belgium

## Abstract

**Introduction:**

Altered pharmacokinetics (PK) in critically ill patients can result in insufficient serum β-lactam concentrations when standard dosages are administered. Previous studies on β-lactam PK have generally excluded the most severely ill patients, or were conducted during the steady-state period of treatment. The aim of our study was to determine whether the first dose of piperacillin-tazobactam, ceftazidime, cefepime, and meropenem would result in adequate serum drug concentrations in patients with severe sepsis and septic shock.

**Methods:**

Open, prospective, multicenter study in four Belgian intensive care units. All consecutive patients with a diagnosis of severe sepsis or septic shock, in whom treatment with the study drugs was indicated, were included. Serum concentrations of the antibiotics were determined by high-pressure liquid chromatography (HPLC) before and 1, 1.5, 4.5 and 6 or 8 hours after administration.

**Results:**

80 patients were treated with piperacillin-tazobactam (n = 27), ceftazidime (n = 18), cefepime (n = 19) or meropenem (n = 16). Serum concentrations remained above 4 times the minimal inhibitory concentration (T > 4 × MIC), corresponding to the clinical breakpoint for *Pseudomonas aeruginosa *defined by the European Committee on Antimicrobial Susceptibility Testing (EUCAST), for 57% of the dosage interval for meropenem (target MIC = 8 μg/mL), 45% for ceftazidime (MIC = 32 μg/mL), 34% for cefepime (MIC = 32 μg/mL), and 33% for piperacillin-tazobactam (MIC = 64 μg/mL). The number of patients who attained the target PK profile was 12/16 for meropenem (75%), 5/18 for ceftazidime (28%), 3/19 (16%) for cefepime, and 12/27 (44%) for piperacillin-tazobactam.

**Conclusions:**

Serum concentrations of the antibiotic after the first dose were acceptable only for meropenem. Standard dosage regimens for piperacillin-tazobactam, ceftazidime and cefepime may, therefore, be insufficient to empirically cover less susceptible pathogens in the early phase of severe sepsis and septic shock.

## Introduction

Severe sepsis and septic shock remain a major cause of morbidity and mortality in medical and surgical ICUs [1]. Although early and appropriate antibacterial therapy is considered a priority in the management of patients with sepsis [2,3], there is evidence that optimizing antibiotic dosage regimens to achieve therapeutic concentrations in the blood and at the site of infection is equally important [4].

Antibiotherapy in critically ill septic patients usually consists of a broad-spectrum β-lactam combined with a glycopeptide and/or an aminoglycoside [5]. These drugs cover a large variety of pathogens and can be empirically used for Gram-negative bacterial infections, including those caused by *Pseudomonas aeruginosa*. The activity of β-lactams is predominantly time-dependent and requires serum and tissue antibiotic concentrations above the minimal inhibitory concentration (MIC) of the pathogen to achieve adequate bacterial killing [6]. This effect is independent of peak levels and there is no significant post-antibiotic effect, except for carbapenems. Clinical data suggest that maximum killing of bacteria occurs when serum concentrations are maintained above the MIC of the causative pathogens for extended periods [7,8]; this may be especially appropriate in patients with compromised host-defences, including critically ill patients [9,10]. However, in conventional bolus dosing regimens, serum β-lactam concentrations may fall to low levels between doses [11,12], with potentially negative effects on clinical response and emergence of resistances.

Antibiotic dosage regimens used in ICU patients are often based on pharmacokinetic (PK) data that were obtained in healthy volunteers or less severely ill patients. Moreover, they rarely take into account the dynamic changes of the septic process that can reduce the efficacy of anti-infective treatments and consequently affect patient outcomes [6]. During severe sepsis and septic shock, increased volume of distribution (Vd) and cardiac output can reduce serum drug concentrations, whereas decreased protein binding and end-organ dysfunction induce higher antibiotic levels [13]. Optimizing antibiotic dosage strategy should involve PK parameters, but therapeutic drug monitoring is necessary in septic patients because large inter-individual PK variations make it difficult to predict antibiotic levels [14]. As previous PK studies on β-lactams in ICU patients have excluded the most severely ill patients or were conducted in the steady-state period of treatment [15-17], the main objective of this study was to determine whether the currently recommended first dose of four broad-spectrum β-lactams (piperacillin-tazobactam, ceftazidime, cefepime, and meropenem) provide adequate plasma concentrations in critically ill septic patients in the ICU. We also tried to determine whether clinical or hemodynamic parameters could affect the PK profile of these drugs during such severe infections.

## Materials and methods

### Study design, patients, antibiotic treatment and data collection

This was a prospective, multicenter, observational study performed in four Departments of Intensive Care in Belgium (at the St-Luc Hospital, Erasme Hospital, and UZ-VUB in Brussels and St Pierre Hospital in Ottignies). The study protocol was approved by the university ethics committees of the different hospitals. Before enrolment, written consent was obtained from the patient or their nearest relative.

All patients admitted to one of the four ICUs between January 2005 and July 2006 were considered for inclusion. Inclusion criteria were a diagnosis of severe sepsis or septic shock [18], either at admission or during the ICU stay, and treatment with a broad-spectrum β-lactam antibiotic (ceftazidime, cefepime, piperacillin-tazobactam, or meropenem). Patients meeting one of the following criteria were excluded: age less than 18 years or more than 85 years; pregnancy or lactation; previous administration of any of the investigated antibiotics; chronic renal failure requiring dialysis; or allergy to any of the investigated antibiotics. The study period was limited to the first 24 hours of antibiotherapy.

Administration of the four β-lactams was made according to local guidelines. These drugs are generally used in the participating centers to treat hospital- or ICU-acquired infections or in the case of community-acquired infection when a more-resistant pathogen may be involved (recent hospitalization or antibiotic therapy, previous colonization by more resistant strain). Piperacillin-tazobactam was preferred as first-line therapy in cases of proven or suspected intra-abdominal infections. Ceftazidime and cefepime was used as first-line therapy in other cases. Meropenem was used as second-line therapy (i.e. failure of piperacillin-tazobactam or cephalosporins) or in case of suspected or previous colonization by extended spectrum beta-lactamase Gram-negative bacteria.

In all study patients, demographics, pre-existing chronic diseases, admission diagnosis and biological data were collected in institutional databases. The severity of illness of each patient was characterized using the Acute Physiology and Chronic Health Evaluation (APACHE) II [19] and sequential organ failure assessment (SOFA) [20] scores determined on the first day of antibiotic treatment. Creatinine clearance (CrCl) was calculated using a standard formula [21]. Treatment of patients with catecholamines, mechanical ventilation, hemofiltration or hemodialysis was recorded, as was length of ICU and hospital stay, overall mortality, and cause of death. Hemodynamic data were collected at baseline, and 8 and 24 hours after the start of the protocol.

### Analytic method for β-lactams

All the patients included in the study received a first dose of 2 g ceftazidime or cefepime, 4 g/0.5 g piperacillin-tazobactam, or 1 g meropenem. The usual daily doses of these antibiotics and dose adjustments for renal function are presented in Additional file 1. All the patients also received amikacin and the two antibiotics were administered simultaneously over 30 minutes using an infusion pump. Blood samples of 5 mL were collected without anticoagulant immediately before the infusion (0 hour) and 1, 1.5, 4.5, and 6 or 8 hours (depending on the frequency of administration of the β-lactam) thereafter; these blood-draw time points were chosen as they belong to the elimination phase of all four antibiotics. The exact sampling time was recorded by the nursing or medical staff. Blood samples were centrifuged at 4000 g for 10 minutes after blood clotting. To allow for possible drug instability at room temperature, serum samples were stored at -80°C until analysis.

All antibiotic quantitative analyses were performed in a centralized reference laboratory (St Luc Hospital). Importantly, as the PK of piperacillin and tazobactam are highly correlated [22], we only measured piperacillin levels. Serum β-lactam concentrations were determined by high-performance liquid chromatography with diode array detection. The intravenous antibiotic formulations were reconstituted according to the manufacturers' recommendations and diluted in water in order to reach stock solution aliquots of 1 mg/mL, stored at -20°C. Before each assay, a fresh calibration curve was prepared from the stock solution and blank serum at the following concentrations: 0.75, 1, 2, 5, 10, 25, and 50 μg/mL for piperacillin-tazobactam; 5, 10, 25, 50, and 100 μg/mL for ceftazidime; 0.1, 0.25, 0.5, 1, 5, 10, 25, and 50 μg/mL for cefepime or meropenem. The calibration and liquid-liquid extraction procedures as well as chromatographic conditions have been described previously [23]. The validation of the four analytical methods was performed over a three-day period with five calibration curves per day (i.e., 15 serum samples per concentration level). All methods were validated according to the published acceptance criteria for specificity, linearity, accuracy, precision (intra-day (repeatability), inter-day (intermediate precision)) and sensitivity (limit of detection (LOD) and limit of quantification (LOQ)). Specificity was determined by the ability to identify the β-lactam from its characteristic retention time and ultraviolet spectrum, and the fine resolution of its chromatographic peak. The linearity of the calibration curve was demonstrated by a significant linear regression analysis, with a determination coefficient (r^2^) more than 0.99. Accuracy was expressed as the percent deviation of the mean observed concentration from the theoretical value, which should not exceed 15%, except at the LOQ (20%). Precision was acceptable if the intra- and inter-day coefficients of variation (CV) were 20% or less at the LOQ and 15% or less at all other concentrations. LOQ was defined as the lowest concentration of the calibration curve, which could be reliably differentiated from background noise with a signal-to-noise ratio of at least 10:1 and quantified with acceptable accuracy (80 to 120%) and precision (CV ≤ 20%); LOD was defined as the lowest concentration that could be detected and reliably differentiated from background noise with a signal-to-noise ratio of at least 3:1.

The carry-over effect was tested by injecting regular blank samples and ultrapure water into the high-performance liquid chromatography system after high concentration calibrators. Under the described chromatographic conditions, piperacillin-tazobactam, ceftazidime, cefepime, and meropenem were identified by sharp and well-resolved peaks. The linearity was statistically confirmed over the concentration range tested for each β-lactam and was associated with an r^2 ^of more than 0.999. The four analytical methods were accurate and precise. LOD and LOQ were 0.50 and 0.75 μg/mL, respectively, for piperacillin-tazobactam, 2.00 and 5.00 μg/mL for ceftazidime, and 0.07 and 0.10 μg/mL for cefepime and meropenem. Appropriate dilution was performed for clinical samples with concentrations above the upper analytic range (corresponding to the calibration curve).

### PK analysis

The PK of the four antibiotics was individually assessed using WinNonlin Professional version 5.0.1. software (Pharsight Corporation, Mountain View, CA, USA). A one-compartment model with first-order elimination was selected to fit the data. Investigated PK parameters included maximal serum concentration (Cmax, calculated by extrapolation of the elimination phase at the end of the infusion) Vd, total clearance (CL), elimination half-live (t_1/2_) and area under the serum concentration-time curve (AUC). Vd and CL were normalized to the body weight.

### PK end-points

The threshold of MIC required for maximal β-lactam activity is still controversial. In this study, the adequacy of β-lactam therapy was assessed by calculating the time spent greater than four times the target MIC (T > 4 × MIC). For each drug, the optimal T > 4 × MIC was considered as: above 50% for piperacillin-tazobactam, above 70% for ceftazidime and cefepime, and above 40% for meropenem in Gram-negative bacterial infections [24]. As the dosage interval of β-lactams is prolonged in renal impairment [see Additional file 1], we calculated the time before the subsequent administration according to the adjustment of the drug regimen to CrCl for each patient. As there is a large variance in MIC values for different bacteria, we considered the MICs for problematic pathogens, such as *P. aeruginosa*, commonly isolated in ICU patients, as the empiric target threshold [25]. Sensitivity thresholds of MIC for this pathogen, as defined by European Committee on Antimicrobial Susceptibility Testing (EUCAST), are: 8 μg/mL or less (ceftazidime, cefepime), 16 μg/mL or less (piperacillin-tazobactam), and 2 μg/mL or less (meropenem) [see Additional file 1] [26]. We, therefore, classified each patient as having an 'adequate' or 'inadequate' PK profile according to the percentage of time during which serum drug concentrations remained more than four times the clinical breakpoint for *P. aeruginosa *(% T > 4 × MIC): 32 μg/mL or more (ceftazidime, cefepime), 64 μg/mL or more (piperacillin-tazobactam), and 8 μg/mL or more (meropenem). Finally, by simulation using the PK parameters of our population, we calculated the probability of achieving target T > 4 × MIC values for other MICs that can be found in ICU-isolated Gram-negative bacteria.

### Statistical analysis

Statistical analyses were performed using the SPSS 13.0 for Windows NT software package (SPSS Inc. 2004, Chicago, IL, USA). Descriptive statistics were computed for all study variables. A Kolmogorov-Smirnov test was used, and histograms and normal-quantile plots were examined to verify the normality of distribution of continuous variables. Discrete variables were expressed as counts (percentage) and continuous variables as means ± standard deviation or median (25th to 75th percentiles). Demographics and clinical differences between study groups were assessed using a chi-squared, Fisher's exact test, Student's t-test, or Mann-Whitney U test, as appropriate. The Pearson's (r) correlation coefficient was used to determine linear correlation as appropriate. Association between variables was tested by simple regression analysis and coefficient of determination (R^2^) in case of non-linear correlation. A *P *< 0.05 was considered as statistically significant.

## Results

### Patient characteristics

We enrolled 80 patients (mean age 63 years, 64% male; Table 1) in the current study. Fifty-five (69%) of the patients were medical admissions and 55 had a hospital or ICU-acquired infection; 58 (72%) had septic shock. The median APACHE II score was 22 and the median SOFA score on admission was 8. Fifty-seven patients (71%) were treated with mechanical ventilation; 22 patients (27%) had acute renal failure. Overall ICU mortality was 38%, mostly due to sepsis. Most infections were respiratory or abdominal and were microbiologically documented in 56 patients (70%). Blood cultures were positive in 32 patients (40%). Forty (50%) cases of sepsis were secondary to Gram-negative bacilli, with 36 infections due to difficult-to-treat pathogens (*P. aeruginosa *(n = 18); *Enterobacter *species (n = 11); *Citrobacter freundii*, *Hafnia alvei *and *Morganella morganii *(n = 2 for each); *Serratia marcescens *(n = 1)).

**Table 1:** Characteristics, hemodynamic and biological data on admission and fluid balance during the first 24 hours (n = 80)

Age (years)	63 ± 13
Male/female	51/29
Body mass index	24.8 ± 4.8
APACHE II on admission	22 (18-28)
SOFA on admission	8 (5-10)
Medical/surgical	55/25
COPD	15 (19%)
Diabetes	21 (26%)
Heart disease	30 (38%)
Chronic renal insufficiency	7 (9%)
Liver cirrhosis	12 (15%)
Immunosuppressive drugs	26 (33%)
Malignancy	26 (33%)
Community/hospital infections	25/55
Severe sepsis/septic shock	22/58
Mechanical ventilation	57 (71%)
Acute renal failure	22 (27%)
ICU stay (days)	12 (5-25)
Overall ICU mortality	30 (38%)
Fluid balance (mL/24 h)	2559 ± 2010
Mean IN (mL/24 h)	4449 ± 1877
Mean OUT (mL/24 h)	1890 ± 1538

Data are expressed as counts (percentage), median (interquartile range) or mean ± standard deviation.

APACHE, Acute Physiology and Chronic Health Evaluation; COPD, Chronic obstructive pulmonary disease; SOFA, Sequential Organ Failure Assessment.

### Pharmacokinetic data

Of the 80 patients, 16 were treated with meropenem, 18 with ceftazidime, 19 with cefepime, and 27 with piperacillin-tazobactam. The mean PK parameters for the four drugs are shown in Table 2. There was marked inter-individual variation in all PK parameters; Vd was increased for all four drugs when compared with healthy volunteers, with consequently a lower Cmax [see Additional file 1]. The median total CL was also reduced when compared with the median CL in healthy volunteers. The median percentage of T > 4 × MIC was 57% for meropenem, 45% for ceftazidime, 34% for cefepime, and 33% for piperacillin-tazobactam (Table 3). Thirteen patients had plasma concentrations less than four times the target MIC after only 90 minutes (ceftazidime = 1; cefepime = 1; piperacillin-tazobactam = 11). The number of patients who attained the target percentage T > 4 × MIC was 12 of 16 for meropenem (75%), 5 of 18 for ceftazidime (28%), 3 of 19 (16%) for cefepime, and 12 of 27 (44%) for piperacillin-tazobactam.

**Table 2:** Pharmacokinetic parameters of the β-lactams

	Vd (L/kg)	Cmax (μg/mL)	AUC (mg.h/mL)	CL (mL/min.kg)	t_1/2 _(hours)
**Piperacillin-tazobactam (n = 27)**	0.38 (0.29-0.43)	123 (72-179)	469 (196-896)	2.02 (1.33-4.26)	2.58 (1.51-3.84)
**Meropenem (n = 16)**	0.43 (0.31-0.77)	35 (29-46)	132 (91-179)	1.87 (1.23-2.63)	2.05 (1.66-3.36)
**Ceftazidime (n = 18)**	0.48 (0.36-0.71)	63 (48-78)	522 (392-634)	0.89 (0.63-1.34)	5.84 (4.13-7.39)
**Cefepime (n = 19)**	0.36 (0.33-0.44)	68 (51-86)	310 (234-422)	1.26 (1.07-1.95)	3.37 (2.26-5.34)

Data are expressed as median [range].

AUC, area under the curve; CL, total clearance; Cmax, peak concentration; t_1/2_, elimination half-time; Vd, volume of distribution.

**Table 3:** Adequate concentrations of the four drugs, with regard to renal dysfunction

	meropenem (n = 16)	ceftazidime (n = 18)	cefepime (n = 19)	piperacillin-tazobactam (n = 27)
**T > 4 × MIC (%)**	57 (25-100)	45 (8-100)	34 (10-100)	33 (0-100)
**Adequate PK, n (%)**	12 (75)	5 (28)	3 (16)	12 (44)
*CrCl <50 mL/min (%)*	5/6 (83)	3/9 (33)	2/12 (17)	10/14 (71)
*CrCl >50 mL/min (%)*	7/10 (70)	2/9 (22)	1/7 (14)	2/13 (15) *

Data are expressed as counts (percentage) or median (range).

CrCl, creatinine clearance; MIC, minimal inhibitory concentration; PK, pharmacokinetic.

* *P *= 0.03 (vs. CrCl < 50 mL/min).

Drug regimens were adapted because of renal impairment in 41 patients (6 treated with meropenem, 9 with ceftazidime, 12 with cefepime, and 14 with piperacillin-tazobactam). The CrCl was similar among the four groups (piperacillin-tazobactam 56 (ranges: 13 to 164) mL/min; meropenem 64 (22 to 134) mL/min; ceftazidime 58 (15 to 145) mL/min; cefepime 40 (13 to 150) mL/min). In patients with renal dysfunction (CrCl <50 mL/min), 5 of 6 (83%) attained the target concentration for meropenem, 3 of 9 (33%) for ceftazidime, 2 of 12 (17%) for cefepime, and 10 of 14 (71%) for piperacillin-tazobactam. For piperacillin-tazobactam, but not for the other antibiotics, patients with renal dysfunction had a significantly higher probability of having adequate drug concentrations than patients with normal renal function (10 of 14 vs. 2 of 13, *P *= 0.03). Calculating the probability of target T > 4 × MIC attainment for several MICs, values more than 90% were obtained for ceftazidime and piperacillin-tazobactam with MIC of 2 μg/mL or less and for cefepime and meropenem with MIC of 1 μg/mL or less (Table 4).

**Table 4:** Probability of target T >4 × MIC attainment for various MICs

		Adequate PK N (%)			
		
MIC (μg/mL)	Target concentration (μg/mL)	meropenem (n = 16)	ceftazidime (n = 18)	cefepime (n = 19)	piperacillin-tazobactam (n = 27)
**32**	**128**	0	0	0	1 (4)
**16**	**64**	0	0	1 (5)	**12 (44)**
**8**	**32**	0	**5 (28)**	**3 (16)**	15 (56)
**4**	**16**	3 (18)	14 (78)	7 (36)	21 (78)
**2**	**8**	**12 (75)**	18 (100)	15 (79)	25 (93)
**1**	**4**	15 (94)	18 (100)	17 (90)	27 (100)
**0.5**	**2**	16 (100)	18 (100)	19 (100)	27 (100)

Data are expressed as counts (percentage). In bold: MIC corresponding to European Committee on Antimicrobial Susceptibility Testing (EUCAST) clinical breakpoints for *Pseudomonas aeruginosa*.

MIC, minimal inhibitory concentration; PK, pharmacok inetics.

### Correlation with clinical variables

No correlation was found between the T > 4 × MIC and any hemodynamic or clinical variable for any of the four drugs, including age, mechanical ventilation, APACHE II or SOFA score at admission, presence of shock, maximum dose of vasopressor agents or fluid balance. There was a significant correlation between CrCl at admission and CL for all drugs (data not shown).

## Discussion

In this study, we show that current standard first doses of piperacillin-tazobactam, cefepime and ceftazidime are insufficient to maintain therapeutic serum concentrations greater than four times the MIC of *P. aeruginosa *in patients with severe sepsis and septic shock. Only with meropenem did a large percentage of patients achieve the bactericidal target of at least 40% T > 4 × MIC. Nevertheless, the probability of reaching the target concentration was greater than 90% only for MICs of 1 μg/mL or less for cefepime, and MICs of 2 μg/mL or less for ceftazidime and piperacillin-tazobactam, suggesting that, for all these drugs, insufficient drug concentrations are obtained for pathogens with higher MICs.

Broad spectrum β-lactams are active against most organisms recovered from ICU patients. Because of the emergence of multidrug-resistant strains and the lack of new antibiotics effective against Gram-negative bacteria [27], a more effective use of existing therapies is necessary. *In vivo *animal studies have demonstrated that β-lactams have a slow continuous kill characteristic that is almost entirely related to the time during which concentrations in tissue and serum exceed the MIC (T > MIC) for the infecting organism [28,29]. The time above the MIC required for maximal β-lactam activity may differ depending on the drug as well as on the pathogen [24]. It has been proposed that, in the absence of post-antibiotic effects, the serum concentration of a β-lactam should exceed the MIC for the respective organism for 100% of the dosing interval [30]. However, experimental studies have suggested that maximum killing of bacteria occurs when β-lactam concentrations exceed four to five times the MIC of the infecting pathogen for extended periods [31,32]. For the treatment of infections in humans, optimal β-lactam concentrations are still controversial. Clinical confirmation of the PK parameters needed for optimal β-lactam efficacy is limited because in several studies drug levels were not measured and the patients included had infections caused mostly by sensitive bacteria [33]. In patients treated with cephalosporins, T > MIC of 100% was associated with greater clinical cure and bacteriological eradication than T > MIC less than 100% [9]. However, the bactericidal activity of cephalosporins has also been shown to be optimal at drug concentrations of about four times the MIC [7]. Even if we preferred 4 × MIC as PK end-point in this study, we did not have enough data to compare the efficacy of these two strategies in the human setting, and a prospective study evaluating the different β-lactams concentrations in the treatment of severe infections is necessary.

Studies on serum concentrations of broad-spectrum β-lactams have already reported that drug levels are insufficient in patients with severe infections. Cefepime (2 g every 12 hours) concentrations were more than 70% T > 16 μg/mL in less than half the patients with sepsis [15] and were adequate only for MICs of 4 μg/mL in all eight patients suffering from post-operative infections [34]. Septic patients with normal renal function had serum cefepime and ceftazidime levels less than 32 μg/mL after a few hours in most cases [11,12]. Ceftazidime trough concentrations were below the median MIC of *P. aeruginosa *in more than half of the patients in another study [35]. In only one study, ceftazidime levels were above the MIC of the isolated pathogens for more than 90% of the time interval; however *Pseudomonas *was isolated in only 4 of 16 patients [16]. Finally, piperacillin concentrations were above therapeutic levels (64 μg/mL) for most of the time interval in patients with sepsis [36] or nosocomial pneumonia [37]. However, serum drug concentrations of meropenem were adequate in most of the patients. In severe infections associated with septicemia, mostly after cardiac surgery, meropenem had serum concentrations above 8 μg/mL for at least 50% of the time in patients with normal and those with reduced CrCl [38]. In patients with ventilator-associated pneumonia, mean T > 4 × MIC for *Pseudomonas *was reported as 52% in one study [39] and 46% in another [17].

Nevertheless, most of these previous studies excluded severely ill patients with septic shock and those with an estimated CrCl limiting the generalization of their results to other populations of critically ill patients. The number of patients was also limited and analyses concerned only the steady-state of the disease. Finally, some of these studies used lower than recommended dosage regimens, which are associated with an increased mortality when susceptible pathogens with higher MICs are present [40,41]. Our study focused on a more severe population of patients, suffering from severe sepsis and septic shock, with higher mortality and morbidity rates than less severely ill ICU populations [42]. Importantly, we used recommended β-lactam regimens that have the greatest likelihood of achieving a bactericidal target in nosocomial pneumonia and bloodstream infections due to Gram-negative bacteria [43,44]. Finally, because antimicrobial treatment of sepsis is often initiated empirically, when pathogens and MICs are still unknown, we used as the target MIC the clinical breakpoint defined by EUCAST for *P. aeruginosa*, an organism that is commonly isolated in ICUs and associated with high mortality rates [25]. This strategy could then be extrapolated to other 'difficult-to-treat' pathogens with high susceptibility breakpoints.

The consequences of these low antimicrobial levels may be more cases of therapeutic failure, higher medical costs and greater emergence of resistance [45]. Moreover, low plasma levels can contribute to lower than expected β-lactam concentrations in the extracellular [46], bronchial [47] or peritoneal fluid [48] with potentially reduced antimicrobial delivery to the target tissues. In view of these results, in septic patients, broad-spectrum β-lactams should be administered more frequently than suggested in non-septic patients, or with doses larger than standard regimens to optimize pathogen exposure to bactericidal concentrations of the drugs. Population modeling simulation showed that continuous or extended β-lactam infusions are required to obtain adequate serum concentrations [45]. However, clinical data that have shown a better outcome using this strategy have come just from retrospective studies in ICU populations with pneumonia [49,50]. Further studies are needed in ICU patients to assess the influence on morbidity and mortality of a strategy whereby antibiotic therapy is selected based on the optimal PK, especially in patients with sepsis and in infections caused by multiresistant pathogens.

Although a relation between the intensity of the septic process and PK abnormalities can be assumed, we did not find any relation between T > 4 × MIC and any demographic, clinical, hemodynamic or biological variables. This finding may be related to the fact that the PK analyses were performed during the early phase of sepsis. Also, as a first dose of antibiotic is largely influenced by Vd, the increased distribution volume may play a key role in reducing antimicrobial concentrations in this setting whereas drug clearance remains the main determinant for drug concentrations at steady-state [13]. CrCl and drug CL showed good correlation, as elimination of the studied drugs is largely dependent on glomerular function [11,38]. Nevertheless, despite a regimen adapted to renal function, patients treated with piperacillin-tazobactam had a higher percentage of adequate concentrations when CrCl was below 50 mL/min. This finding may be related to the complex elimination of piperacillin-tazobactam, which includes biliary excretion [13]. Indeed, the hepatic metabolism of this drug is variable and difficult to measure and most studies on piperacillin-tazobactam PKs in patients with renal failure have included patients with normal hepatic function [51]. It is possible that, as severe sepsis is frequently associated with liver dysfunction, this may have contributed to greater than expected drug accumulation in some patients. Further studies are needed to evaluate the impact of renal and hepatic dysfunction on piperacillin-tazobactam regimens in critically ill patients.

Our study has some limitations. First, we evaluated the PK profile of β-lactams only during the first dose, and thus cannot make any statement with regard to subsequent doses. Vd may decrease during therapy when capillary leakage subsides and sepsis resolves [52]; in such circumstances, coupled with persistent renal dysfunction, standard β-lactam doses may be sufficient to achieve therapeutic concentrations. Second, as only free drug is the active moiety, it has been recommended that all PK/pharmacodynamic indices should be referenced to the unbound (free) fraction of the drug, especially for some drugs such as piperacillin, which has 20 to 30% protein binding [53]. Third, the inadequate PK/pharmacodynamic indices observed in our study should be considered in relation to the empirical MIC target, and may be different with other susceptibility patterns (MIC distributions) of pathogens at individual institutions. Attention should therefore be paid to establish the MIC values of these pathogens in order to adapt dosage regimens. Also, CrCl was estimated using the Cockroft and Gault formula, which shows important limitations in predicting the real CrCl in ICU patients [54]. Finally, the four groups were heterogeneous and, therefore, the numbers may be too small to fully reflect the characteristics of the drugs in this setting. However, an important concern is the highly variable and unpredictable inter-individual PKs for cephalosporins and piperacillin and whether these drugs can be considered an appropriate agent to use as initial empirical therapy for critically ill patients with severe sepsis and septic shock, particularly in those with potentially less susceptible Gram-negative bacterial strains.

## Conclusions

The treatment of infections in the critically ill patient remains a significant challenge for clinicians. Standard first doses of broad-spectrum β-lactams provided inadequate levels to achieve target serum concentrations for extended periods of time in critically ill patients with sepsis. Improved characterization of the pharmacodynamic properties of these antimicrobials may lead to revisions in recommendations on dosing in severe infections, especially in the early phase of severe sepsis and septic shock.

## Key messages

• Recommended doses of piperacillin-tazobactam, cefepime and ceftazidime provided serum drug concentrations during the first 24 hours of treatment that were insufficient to cover *P. aeruginosa *and other less susceptible bacteria in patients suffering from severe sepsis and septic shock.

• Recommended doses of meropenem resulted in adequate concentrations to cover *P. aeruginosa *and other less susceptible bacteria in 75% of patients.

• In patients treated with piperacillin-tazobactam, renal dysfunction is associated with a better adequacy of drug concentrations compared with normal renal function.

• Therapeutic drug monitoring is necessary to optimize β-lactam concentrations as no clinical or biological variable can predict β-lactam concentrations in this population.

## Abbreviations

APACHE: acute physiology and health evaluation; AUC: area under the curve; CL: total clearance; Cmax: peak concentration; CrCl: creatinine clearance; CV: coefficient of variation; EUCAST: European Committee on Antimicrobial Susceptibility Testing; LOD: limit of detection; LOQ: limit of quantification; MIC: minimum inhibitory concentration; PK: pharmacokinetics; SOFA: sequential organ failure assessment; t_1/2_: elimination half-time; Vd: volume of distribution.

## Competing interests

FST, FJ, JLV, TD and PFL have received honoraria for lectures from Astra Zeneca. JLV is also on the speakers list of GlaxoSmithKline. The other authors declare that they have no competing interests.

## Authors' contributions

FJ and PFL conceived the study protocol. FST, FJ, PFL, TD, XW, BL and HS participated in the design and coordination of the study. ID and PW performed the PK analyses. FST, PFL, DDB, JLV and FJ drafted the present manuscript. All authors read and approved the final manuscript.

## Supplementary Material

Additional file 1 **Three tables showing usual daily doses of antibiotics and dose adaptation to renal function, Minimum inhibitory concentrations (MICs) for *Pseudomonas aeruginosa *and *Enterobacteriaceae *according to European Committee on Antimicrobial Susceptibility Testing (EUCAST); and mean pharmacokinetic parameters in healthy volunteers**. Click here for file
