# Supplementary material for: Insufficient β-lactam concentrations in the early phase of severe sepsis and septic shock
Source: Crit Care. 2010 Jul 1;14(4):R126. doi: 10.1186/cc9091 (PMC2945087; doi:10.1186/cc9091)
Supplement: Additional file 1 — Three tables showing usual daily doses of antibiotics and dose adaptation to renal function, Minimum inhibitory concentrations (MICs) for Pseudomonas aeruginosa and Enterobacteriaceae according to European Committee on Antimicrobial Susceptibility Testing (EUCAST); and mean pharmacokinetic parameters in healthy volunteers. [file cc9091-S1.doc]

**Additional data file**

**Table 1.** Usual daily doses of antibiotics and dose adaptation to renal function.

| **CrCl** | Daily Dose | | | | |
| --- | --- | --- | --- | --- | --- |
|  | **> 80 mL/min** | **50-80 mL/min** | **10-50 mL/min** | **<10 mL/min** | **CRRT** |
| CAZ | 2g q8h | 2g q12h | 1g q12h | 0.5g daily | 2g q12h |
| **CEF** | 2g q8h | 2g q12h | 1g q12h | 0.5g daily | 2g q12h |
| **PIP** | 4g q6h | 4g q6h | 4g q8h | 4g q12h | 4g q8h |
| **MERO** | 1g q8h | 1g q12h | 0.5g q12h | 0.5g daily | 1g q12h |

**Table 2.** Minimal Inhibitory Concentrations (MICs) for *Pseudomonas aeruginosa* and *Enterobacteriaceae* according to EUCAST.

|  | ***Enterobacteriaceae*** | | ***P.aeruginosa*** | |
| --- | --- | --- | --- | --- |
|  | **S** | **R** | **S** | **R** |
| CAZ | 1 | 8 | 8 | 8 |
| **CEF** | 1 | 8 | 8 | 8 |
| **PIP** | 8 | 16 | 16 | 16 |
| **MERO** | 2 | 8 | 2 | 8 |

**Table 3.** Mean pharmacokinetic parameters in healthy volunteers.

|  | **Vd (L/kg)** | **Cmax (μg/mL)** | **CL (mg.h/mL)** | **t _½_ (h)** |
| --- | --- | --- | --- | --- |
| CAZ | 0.25 | 165 | 1.60 | 2 |
| **CEF** | 0.2 | 150 | 1.90 | 2 |
| **PIP** | 0.15 | 425 | 2.38 | 1.1 |
| **MERO** | 0.25-0.3 | 50 | 3.62 | 1 |

From:

Kim MK, Capitano B, Mattoes BH et al. Pharmacokinetic and pharmacodynamic evaluation of two dosing regimens for piperacillin-tazobactam. Pharmacotherapy 2002 ; 22 :569-577

Van der Auwera P, Santella PJ. Pharmacokinetics of cefepime: a review. J Antimicrob Chemother 1993; 32 :103-115

[Kuti JL](http://www.ncbi.nlm.nih.gov/pubmed?term="Kuti JL"%5BAuthor%5D&itool=EntrezSystem2.PEntrez.Pubmed.Pubmed_ResultsPanel.Pubmed_RVAbstract), [Horowitz S](http://www.ncbi.nlm.nih.gov/pubmed?term="Horowitz S"%5BAuthor%5D&itool=EntrezSystem2.PEntrez.Pubmed.Pubmed_ResultsPanel.Pubmed_RVAbstract), [Nightingale CH](http://www.ncbi.nlm.nih.gov/pubmed?term="Nightingale CH"%5BAuthor%5D&itool=EntrezSystem2.PEntrez.Pubmed.Pubmed_ResultsPanel.Pubmed_RVAbstract), [Nicolau DP](http://www.ncbi.nlm.nih.gov/pubmed?term="Nicolau DP"%5BAuthor%5D&itool=EntrezSystem2.PEntrez.Pubmed.Pubmed_ResultsPanel.Pubmed_RVAbstract). Comparison of pharmacodynamic target attainment between healthy subjects and patients for ceftazidime and meropenem. Pharmacotherapy 2005 Jul;25(7):935-41

EUCAST. Rationale documents for EUCAST. Available at: [*http://www.eucast.org/*](http://www.eucast.org/) *documents/rd/.* Accessed March 2010

*Abbreviations*:

CAZ = ceftazidime; CEF = cefepime; PIP = piperacillin; MERO = meropenem; Vd = distribution volume; Cmax = peak concentration; CL = total clearance; t _½_ = half-life time; CrCl = creatinine clearance; CRRT = continuous renal replacement therapy
